# A case of renal hypouricemia type 1 associated with compound heterozygous *URAT1/SLC22A12* variants A209V and F379L: clinical evidence for their pathogenic significance

**DOI:** 10.1007/s13730-026-01189-0

**Published:** 2026-10-12

**Authors:** Naoyuki Otani, Marie Yamamoto, Tomoaki Takata, Shinobu Sugihara, Masanari Kuwabara, Masaharu Fukuki, Yusuke Endo, Seiko Shimizu, Akiyoshi Nakayama, Akihiro Otsuki, Hirotaka Matsuo, Kimiyoshi Ichida, Ichiro Hisatome

**Affiliations:** 1 Department of Cardiology, Dokkyo Medical University Nikko Medical Center, 145-1 Moritomo, Nikko City, Tochigi 321-1298 Japan; 2 Department of Cardiology, NHO Yonago Medical Center, Yonago, Japan; 3 Department of Nephrology, NHO Yonago Medical Center, Yonago, Japan; 4 Division of Gastroenterology and Nephrology, Faculty of Medicine, Tottori University, Yonago, Japan; 5 Matsue Health Service Center, Shimane University, Matsue, Japan; 6 Division of Public Health, Center for Community Medicine, Jichi Medical University, Shimotsuke, Japan; 7 Division of Cardiovascular Medicine, Department of Medicine, Jichi Medical University, Shimotsuke, Japan; 8 Organization for Research Initiative and Promotion, Tottori University, Yonago, Japan; 9 Department of Integrative Physiology and Bio-Nano Medicine, National Defense Medical College, Tokorozawa, Saitama Japan; 10 International Research Collaboration Officer, National Defense Medical College Research Institute, National Defense Medical College, Tokorozawa, Japan; 11 Division of Anesthesiology and Critical Care Medicine, Department of Surgery, Tottori University Faculty of Medicine, Yonago, Japan; 12 School of Pharmacy, Tokyo University of Pharmacy and Life Sciences, Tokyo, Japan; 13 Division of Nephrology and Hypertension, Department of Internal Medicine, The Jikei University School of Medicine, Tokyo, Japan; 14 Chiba Health Promotion Center, East Japan Railway Company, Chiba, Japan; 15 Department of Cardiology, Matsue City Hospital, Matsue, Japan

**Keywords:** Renal hypouricemia, *URAT1*, Compound heterozygosity, Nephrolithiasis, CKD

## Abstract

Renal hypouricemia (RHUC) is an inherited renal tubular disorder characterized by low serum uric acid levels and excessive urinary uric acid excretion due to impaired urate reabsorption in proximal tubules. Type 1 RHUC (RHUC1) is caused by pathogenic variants in *URAT1/SLC22A12*. In Japanese patients, recurrent variants such as W258X and R90H account for a substantial proportion of RHUC1 cases; however, the clinical significance of rare *URAT1* variant combinations remains incompletely understood. A 69-year-old woman was referred to our hospital for evaluation of renal dysfunction and hypouricemia. Laboratory examination revealed severe hypouricemia (serum uric acid, 0.7 mg/dL) and markedly increased urinary uric acid excretion (Cua/Ccr, 60.5%). She had chronic kidney disease (CKD) stage G3b and a renal stone. Genetic analysis identified two heterozygous variants, A209V and F379L, in *URAT1/SLC22A12* gene. The variants were considered likely to represent compound heterozygosity based on the clinical phenotype and their previously reported functional impairment. Although A209V and F379L have individually been reported to impair urate transport, the clinical phenotype associated with this variant combination has not been well characterized. The severe hypouricemia and excessive urinary uric acid excretion observed in this patient provide clinical evidence supporting the pathogenic significance of the A209V and F379L combination. The coexistence of nephrolithiasis and CKD further raises the possibility that excessive urinary urate excretion and stone formation may contribute to renal dysfunction. In addition, the improvement in stone-related urinary abnormalities during potassium citrate treatment suggests that urinary alkalization may be beneficial in the management of nephrolithiasis associated with RHUC.

## Introduction

Renal hypouricemia (RHUC) is an inherited disorder characterized by both low serum uric acid levels and the excess urinary uric acid excretion resulting from impaired uric acid reabsorption in proximal tubules. The disorder is primarily caused by loss-of-function variants in *URAT1/SLC22A12* or *GLUT9/SLC2A9* genes [1–4], which encode major uric acid transporters. RHUC caused by pathogenic variants in *URAT1* and *GLUT9* is classified as renal hypouricemia type 1 (RHUC1, OMIM #220150) and renal hypouricemia type 2 (RHUC2, OMIM #612076), respectively. RHUC is relatively common in Japanese populations, with an estimated prevalence of approximately 0.2% in men and 0.4% in women. The disorder has also been recognized in Korean and Roma populations, suggesting ethnic differences in its genetic background [5]. Loss-of-function variants in *URAT1* markedly impair renal uric acid reabsorption, resulting in hypouricemia and excessive urinary uric acid excretion [1, 2, 6, 7]. Population-based genetic studies and in vitro analyses have identified several rare *URAT1* variants that contribute to reduced serum uric acid levels [6, 7]. In general, individuals carrying a single heterozygous *URAT1* variant exhibit only mild reductions in serum uric acid levels, whereas the clinical significance of compound heterozygous variants remains poorly understood. Recent functional studies have suggested that A209V (Exon 3) and F379L (Exon 7) variants impair uric acid transporter activity and may therefore contribute to RHUC [6, 7]. However, whether these variants are sufficient to cause clinically apparent RHUC has not been established.

Here, we report a patient with two heterozygous *URAT1* variants, A209V and F379L, that were considered highly likely to represent compound heterozygosity based on the clinical phenotype. The patient presented with severe renal hypouricemia accompanied by chronic kidney disease (CKD) and nephrolithiasis. This case provides clinical evidence supporting the pathogenic role of this rare variant combination and expands the genotype–phenotype spectrum of RHUC.

## Case presentation

**Fig. 1:**
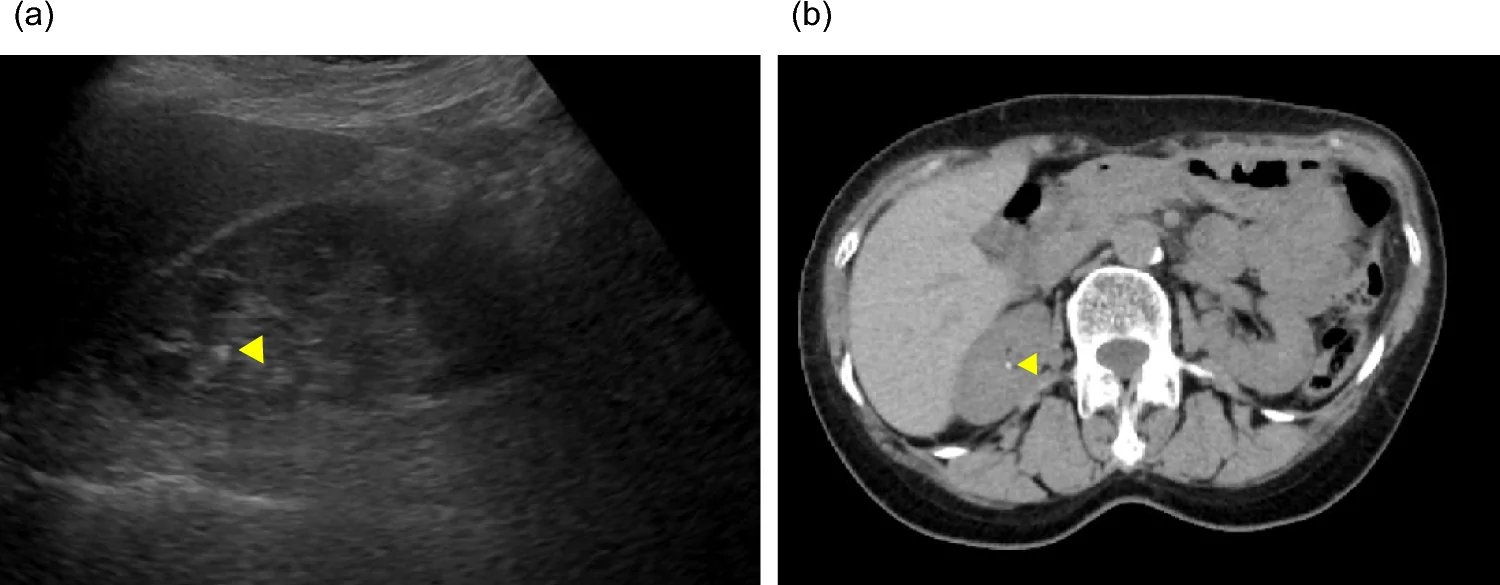
Imaging findings demonstrating urolithiasis in this case. **a** Renal ultrasonography showing a hyperechoic lesion with acoustic shadowing in the left kidney (yellow arrowhead), consistent with a renal calculus. **b** Non-contrast computed tomography demonstrating a high-density lesion in the left kidney (yellow arrowhead), confirming the presence of a renal stone. The mean CT attenuation value of the stone was 102 ± 40 HU

A 69-year-old woman was referred to our hospital by her primary care physician for further evaluation of renal impairment and hypouricemia. Her medical history was notable for dyslipidemia treated with rosuvastatin. She had no history of hypertension or diabetes mellitus. For several years, her serum uric acid levels had persistently remained below 1.0 mg/dL and she had been advised to maintain adequate fluid intake to reduce the risk of exercise-induced acute kidney injury (EIAKI). Longitudinal renal function data available from 2015 showed an overall decline in renal function, with serum creatinine increasing from 0.88 mg/dL in 2015 to 1.17 mg/dL in 2025 and estimated glomerular filtration rate (eGFR) decreasing from 51 to 35.5 mL/min/1.73 m^2^ during the same period. In 2026, serum creatinine was 1.06 mg/dL and eGFR was 39.4 mL/min/1.73 m^2^. No episodes of acute kidney injury were documented. Her family history was notable for hypouricemia in her elder brother who was being followed by a urology clinic. On physical examination, her blood pressure was 137/78 mmHg, pulse rate was 57 beats/min, height was 148.8 cm, and body weight was 42.8 kg. Laboratory findings obtained in October 2023, before initiation of potassium citrate therapy are summarized in Table 1. Serum uric acid level was markedly decreased at 0.7 mg/dL, while serum creatinine was 1.0 mg/dL and eGFR of 42.5 mL/min/1.73 m^2^. Urine pH was 6.0, and urine occult blood was trace-positive. Urinary β_2_-microglobulin was elevated. Urinary uric acid and creatinine concentrations were 44.3 and 104.64 mg/dL, respectively, with a markedly elevated uric acid clearance ratio (Cua/Ccr) of 60.5%. Abdominal ultrasonography and computed tomography revealed a renal stone in the left kidney (Fig. 1A, B).

**Table 1:** Laboratory findings before initiation of potassium citrate therapy

Parameter	Value
White blood cell count	3000/μL
Hemoglobin	13.4 g/dL
Platelet count	165 × 10^3^/μL
C-reactive protein	0.14 mg/dL
Serum creatinine	1.0 mg/dL
eGFR	42.5 mL/min/1.73 m^2^
Serum uric acid	0.7 mg/dL
Urine pH	6.0
Urine specific gravity	1.025
Urine occult blood	Trace
Urinary uric acid	44.3 mg/dL
Urinary creatinine	104.64 mg/dL
Cua/Ccr	60.48%
Urinary β_2_-microglobulin	745 μg/L
Urinary NAG	6.7 IU/L

*eGFR* Estimated glomerular filtration rate; *Cua/Ccr* Uric acid clearance ratio; *NAG* N-acetyl-β-D-glucosaminidase

**Fig. 2:**
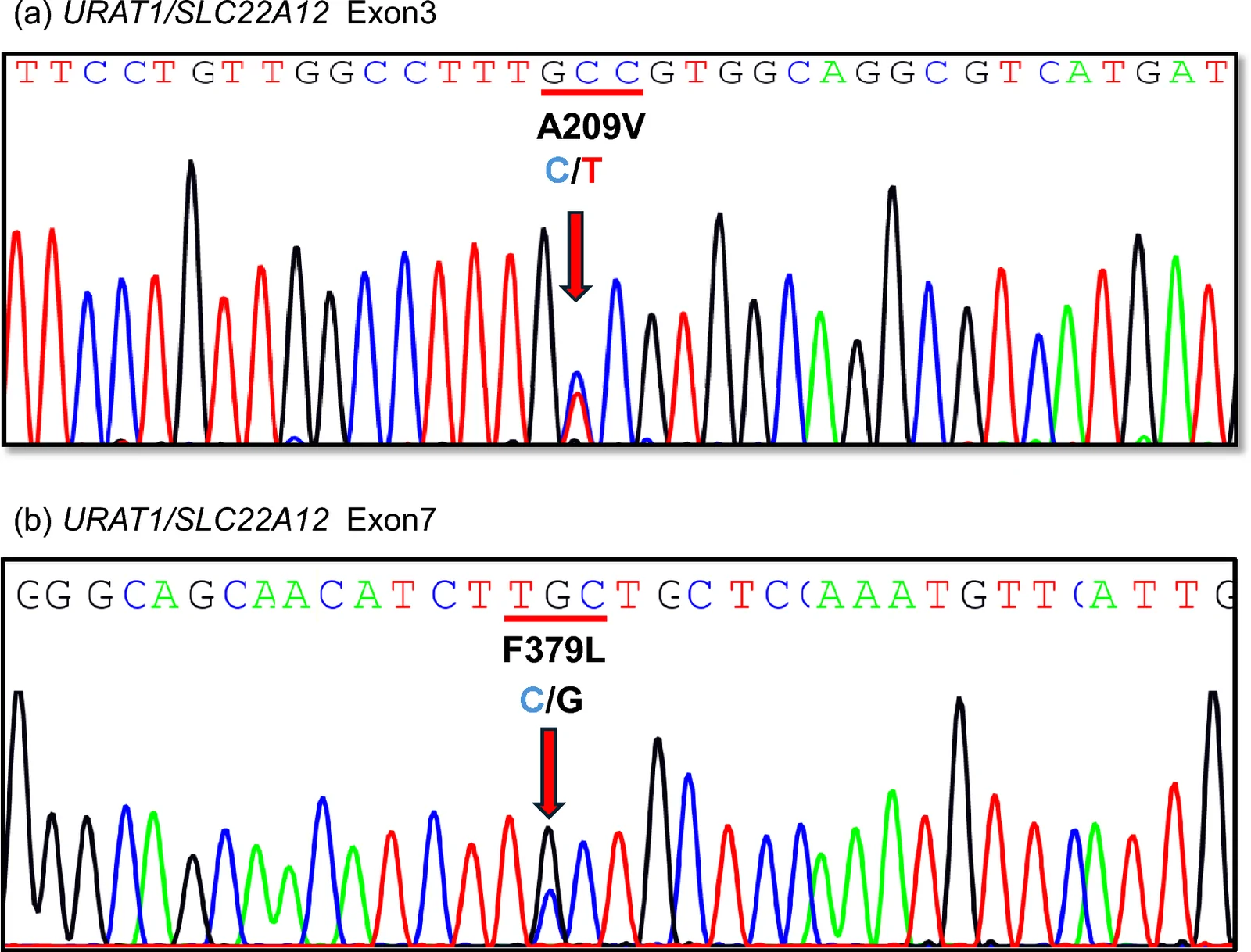
Direct sequencing analysis of the *URAT1/SLC22A12* gene. **a** Electropherogram showing a heterozygous A209V variant in Exon 3. **b** Electropherogram showing a heterozygous F379L variant in Exon 7. Both variants were confirmed by direct sequencing

Genetic analysis was performed after the Institutional Review Boards of Tottori University Hospital and Yonago Medical Center approved the study protocol (approval numbers: 21A048, 040301 and 040611, respectively). Written informed consent was obtained from the patient. Genetic analysis was performed using targeted next-generation sequencing on the Ion GeneStudio S5 (Thermo Fisher Scientific), followed by direct sequencing using the SeqStudio Genetic Analyzer (Thermo Fisher Scientific). The following primers were used: for A209V (rs552232030), forward 5′–CAAGCATAGGGTGGGCTCTAGG–3′ and reverse 5′–GTCCACTCCATCACTGCAAGG–3′; for F379L (rs2039388764), forward 5′–TGCCCCAGGTTCGCCTTTG–3′ and reverse 5′–AGGAGTCCTTCTTGACCCCGTTT–3′. Genetic analysis identified heterozygous A209V and F379L in exon 3 and exon 7, respectively, of the *URAT1*/*SLC22A12* gene (Fig. 2). Based on the clinical and genetic findings, the patient was diagnosed with renal hypouricemia type 1 associated with presumed compound heterozygous *URAT1* variants.

Given the presence of a renal stone and excessive urinary uric acid excretion, treatment with potassium citrate was initiated to alkalize the urine. During treatment, urine pH was maintained at approximately 7.0–7.5, and microscopic hematuria present before treatment resolved. No episodes of renal colic occurred during follow-up. After discontinuation, however, urine pH decreased to as low as 5.5 and microscopic hematuria recurred, suggesting the possibility of stone-related urinary tract abnormalities. Potassium citrate was therefore resumed, after which urine pH stabilized at approximately 7.0 and the hematuria resolved.

## Discussion

The present case had several notable features: (1) severe hypouricemia associated with markedly increased urinary uric acid excretion, (2) the presence of A209V and F379L variants in *URAT1*, (3) coexistence of chronic kidney disease (CKD) and nephrolithiasis, (4) a clinical course suggesting that excessive uric acid excretion may contribute to stone formation and that urinary alkalization may improve stone-related urinary abnormalities.

RHUC is an inherited renal tubular disorder characterized by low serum uric acid levels and the excessive urinary uric acid excretion resulting from impaired uric acid reabsorption in the proximal tubules. In the present case, two heterozygous *URAT1*/*SLC22A12* variants, A209V and F379L, were identified. Although the allelic phase of these variants was not directly determined, the marked clinical phenotype was highly suggestive of presumed compound heterozygosity. Her elder brother also had hypouricemia, suggesting a shared genetic background. RHUC caused by *URAT1* variants exhibits ethnic differences in genotype distribution [5, 6]. Most Japanese patients with RHUC1 carry recurrent variants such as W258X and R90H [8–11], whereas distinct rare variants or founder variants such as R434C, Q382L and T467M have been reported in Korean, Chinese and Roma populations, respectively [5–7, 12]. Recent population genetic studies further suggest that rare *URAT1* variants contribute to the missing heritability of serum urate levels and often show population-specific distributions [6]. Characterization of such variants is therefore important for improving the genetic diagnosis of RHUC in Japanese population. In this context, the present case carrying A209V and F379L expands the genotype–phenotype spectrum of RHUC1 and highlights the possibility that clinically overt RHUC may result from rare combinations of *URAT1* variants captured by first-line genetic screening focused on W258X and R90H [13].

A single heterozygous *URAT1* variant generally causes only a mild reduction in serum uric acid levels and may not result in clinically overt RHUC [11, 14]. In contrast, the present case had severe hypouricemia (serum uric acid level: 0.7 mg/dL) and increased urinary uric acid excretion (Cua/Ccr: 60.5%). In vitro functional studies have demonstrated impaired urate transport associated with A209V and F379L [6, 7]. Although either variant alone in the heterozygous state may be insufficient to cause severe hypouricemia, the coexistence of A209V and F379L may substantially impair renal urate reabsorption. The severe clinical phenotype is consistent with biallelic URAT1 dysfunction; however, because molecular phasing and family segregation analyses were not performed, the possibility that the two variants are located in cis cannot be completely excluded. Nevertheless, the marked hypouricemia, excessive urinary uric acid excretion, and previously demonstrated functional impairment of both variants support the interpretation that presumed compound heterozygosity contributed to the RHUC phenotype. To further interpret the clinical significance of the present case, we descriptively analyzed patients with identified URAT1 variants according to *URAT1* genotype (Table 2). Consistent with this interpretation, patients carrying two *URAT1* variants exhibited severe hypouricemia and increased urinary urate excretion with clinical phenotypes similar to those of patients carrying homozygous *URAT1* variants.

**Table 2:** Clinical characteristics according to *URAT1* genotype status

Genotype group	n	SUA (mg/dL) Median (IQR)	Cua/Ccr (%) Median (IQR)	Uua/Ucr Median (IQR)	Urine pH Median (IQR)	Urolithiasis n (%)	eGFR (mL/min/1.73 m^2^) Median (IQR)
Homozygous variants	3	0.7 (0.6–0.8)	67.1 (65.0–70.4)	0.50 (0.50–0.55)	7.5 (7.0–7.5)	2 (67)	83.1 (68.4–94.2)
Compound heterozygous variants	5	0.6 (0.5–0.7)	46.8 (44.0–52.1)	0.43 (0.42–0.49)	7.0 (5.5–7.0)	1 (20)	90.1 (82.6–95.0)
Heterozygous variants	4	1.4 (1.2–1.5)	26.3 (18.4–40.2)	0.60 (0.57–0.68)	6.5 (5.8–6.5)	0 (0)	101.5 (97.1–107.1)

Individual variants are shown in Table 3. *SUA* Serum uric acid; *Cua/Ccr* Uric acid clearance ratio; *Uur/Ucr* Urinary uric acid to creatinine ratio; *eGFR* Estimated glomerular filtration rate; *IQR* Interquartile range

The mechanism underlying the severe phenotype associated with A209V and F379L variants remains unclear. Functional analyses have shown that the A209V variant protein could localize to the cell membrane, but has markedly reduced uric acid transport activity [6], whereas F379L variant protein exhibits defective membrane trafficking with profound loss of uric acid transport activity [7]. Thus, the coexistence of variants with different functional defects may result in a substantial overall reduction in URAT1-mediated urate reabsorption, accounting for both severe hypouricemia and excessive urinary uric acid excretion.

**Fig. 3:**
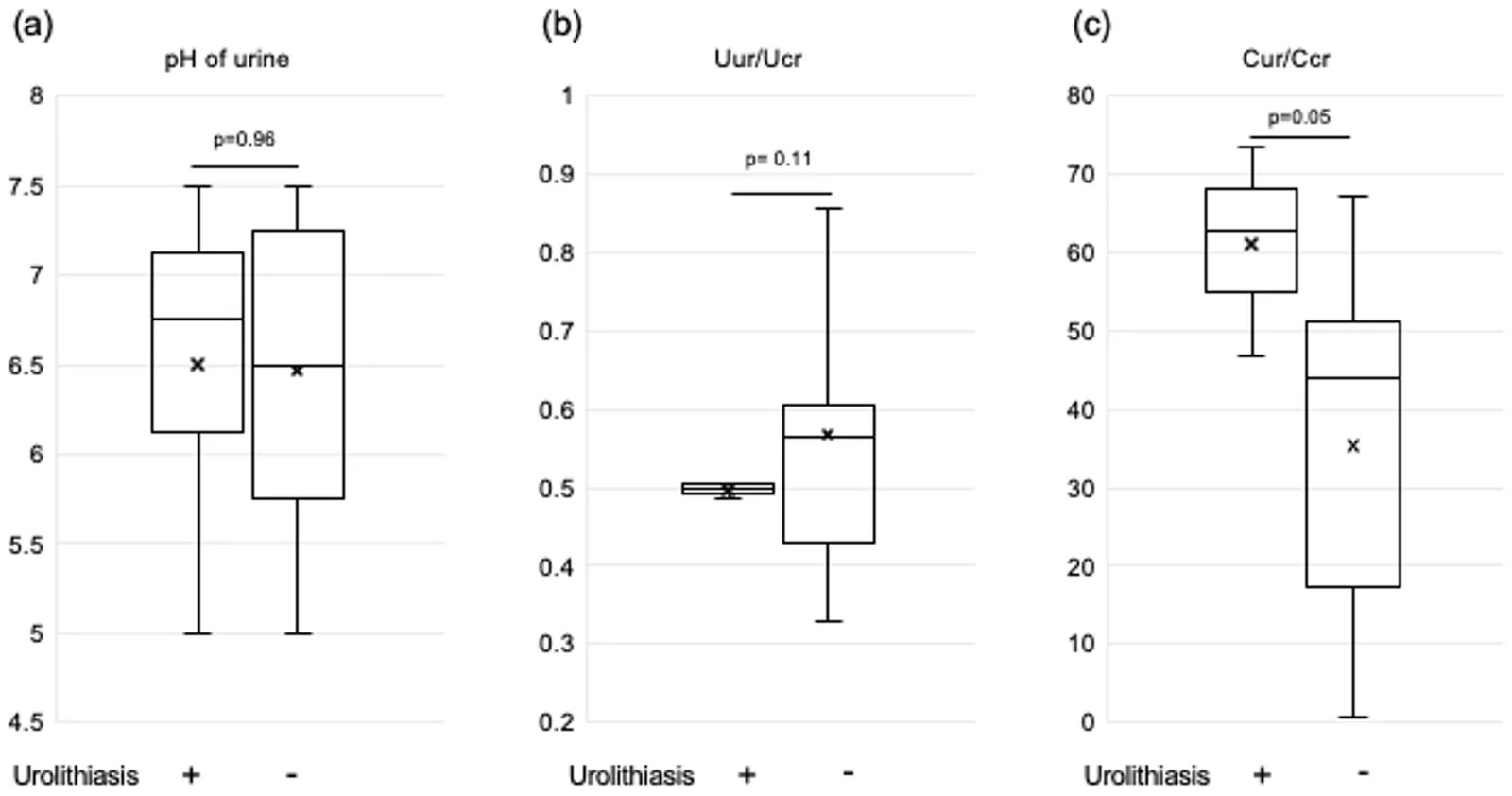
Comparison of urinary uric acid-related parameters between hypouricemic patients with and without urolithiasis. Box plots showing **a** urine pH, **b** urinary uric acid-to-creatinine ratio (Uur/Ucr), and **c** uric acid clearance-to-creatinine clearance ratio (Cua/Ccr). Cua/Ccr was higher in patients with urolithiasis than in those without urolithiasis (*p* = 0.05), whereas urine pH and Uur/Ucr showed no apparent differences between the groups (*p* = 0.96 and 0.11, respectively)

Another important feature of this case was the coexistence of nephrolithiasis and CKD, both recognized complications of RHUC. To explore the relationship between urinary uric acid excretion and nephrolithiasis, we compared patients with RHUC according to the presence and absence of kidney stone (Table 3, Fig. 3). Patients with urinary stones tended to exhibit greater uric acid excretion, particularly higher Cua/Ccr values. Since the stone composition was not directly analyzed, it remains uncertain whether excessive urinary uric acid excretion contributed to the formation of a uric acid stone in the present case. However, non-contrast CT demonstrated a renal calculus with a low mean attenuation value of 102 ± 40 HU. Some study indicated that a significant difference was found between the Hounsfield measurement of uric acid calculi (mean 344 ± 152 HU) and the Hounsfield measurement of calcium oxalate calculi (mean 652 ± 490 HU), which may be compatible with a uric acid–containing stone in the present case [16]. Although uric acid solubility depends on both urine pH and urinary uric acid concentration, neither parameter differed significantly between patients with and without urinary stones in our cross-sectional analysis [17]. However, single measurements of urine pH may not reflect temporal fluctuations relevant to stone formation.

**Table 3:** Clinical and laboratory characteristics of patients with renal hypouricemia according to the presence or absence of urolithiasis

Age	Sex	*URAT1* gene mutation	Complication	urolithiasis	Serum				Urine			Uua/Ucr	Cua/Ccr
					Uric acid	Creatinine	eGFR	pH	Specific gravity	Uric acid	Creatinine		
75	Male	G774A homo	Chronic heart failure, atrial fibrillation, hypertension	+	0.8	1.0	53.8	6.5	1.006	24.7	49.1	0.50	62.9
63	Male	G774A homo	Sjögren’s syndrome	+	0.4	0.6	105.2	7.5	1.009	29.6	59.6	0.50	73.6
84	Female	ND		+	2.7	0.7	58.5	5	1.024		29.6	0.51	
33	Female	V138M/W258X compound hetero		+	0.6	0.6	95.0	7	1.025	52	107.3	0.49	46.8
Median (IQR)					0.7 (0.6–1.3)	0.7 (0.6–0.8)	76.7 (57.3–97.6)	6.8 (6.1–7.1)	1.017 (1.008–1.024)	29.6 (27.2–40.8)	54.4 (44.2 –71.5)	0.50 (0.50–0.50)	62.9 (54.8–68.2)
63	Female	ND	Xanthinuria, hypertension	−			144.1	7	1.007				
44	Female	ND		−	2.5	0.48	109.47	6	1.007	12.7	16.2	0.8	15.1
68	Male	G774A hetero		−	0.7	0.72	84.24			20.8	35.9	0.6	59.6
39	Male	ND	Dyslipidemia	−	0.8	0.94	73.64	5.5	1.014	39.1	91	0.4	50.5
75	Male	Wild type		−	1.8	0.47	96.74	7.5	1.012	141.1	326.8	0.4	11.3
56	Male	ND		−	0.8	0.7	90.27	7.5	1.016	71.1	135.1	0.5	46.1
80	Female	Wild type		−	2.5	0.78	54.02	6.5	1.022	24.9	44.1	0.6	17.6
76	Female	T1289C hetero	Diabetes mellitus	−	1.4	0.44	101.56	5	1.020	16.8	19.6	0.9	18.7
50	Female	G774A hetero		−	1.8	0.5	101.36	6.5	1.025	99.4	159.2	0.6	17.3
66	Male	G774A/G269A compound hetero		−	0.9	1.1	54.24	5.5	1.011	66.7	155.7	0.4	52.4
34	Female	G774A/IVS2+1G compound hetero	Diabetes mellitus	−	0.5	0.31	202.67	7	1.018	83.5	99.4	0.8	52.1
64	Female	C1137G hetero		−	1.3	0.55	123.67	6.5	1.019	66.9	122.8	0.5	33.8
50	Male	Wild type		−	0.8	0.63	104.6	6.5	1.003	19.4	34.3	0.6	44.5
43	Female	G774A/G269A compound hetero		−	0.3	0.57	90.1	7.5	1.017	30	90	0.3	0.6
45	Male	G774A/G774A homo		−	0.7	0.8	83.1	7.5	1.017	71.6	122	0.6	67.1
71	Male	626 T/1137G compound hetero		−	0.7	0.73	82.61	5	1.008	50.9	120.6	0.4	44.0
Median (IQR)					0.8 (0.7–1.6)	0.6 (0.5–0.8)	93.5 (83.0–105.8)	6.5 (5.8–7.3)	1.016 (1,010–1.019)	50.9 (22.9–71.4)	99.4 (40.0–105.8)	0.6 (0.4–0.6)	44.0 (17.5–51.3)

The 20 patients included 11 patients previously reported in [15], one patient previously reported in [17], and eight previously unpublished patients evaluated at Tottori University Hospital and NHO Yonago Medical Center. *eGFR* Estimated glomerular filtration rate; *Uua/Ucr* Urinary uric acid to creatinine ratio; *Cua/Ccr* Uric acid clearance ratio

Given the clinical course suggesting a beneficial effect of urinary alkalization on hematuria in the present case, urinary pH management may contribute to stone prevention. Notably, urine pH was maintained at approximately 7.0–7.5 and microscopic hematuria resolved during potassium citrate treatment in the present patient. After treatment was discontinued, urine pH decreased to as low as 5.5 and microscopic hematuria recurred; both improved after potassium citrate was resumed. Although this clinical course does not establish causality, it suggests that urinary alkalization may be beneficial for reducing stone-related urinary abnormalities in RHUC [18].

The relationship between nephrolithiasis and CKD in RHUC remains unclear. Several cross-sectional studies have reported an association between hypouricemia and renal dysfunction [19, 20] including EIAKI [21–23]. Proposed mechanisms of renal injury include renal vasoconstriction due to impaired nitric oxide-dependent and endothelium-derived hyperpolarizing factor-dependent vasodilation, tubular energy depletion during exercise, and tubular injury and inflammation associated with excessive urinary urate and intratubular urate crystal precipitation [24, 25]. The present case had no documented history of EIAKI, which may be a less likely explanation for her CKD. In our case series (Table 3), the patients with urinary stones tended to have lower eGFR values than those without stones (76.7 vs. 93.5 mL/min/1.73 m^2^, *p* = 0.27). Although this difference was not statistically significant, it raises the possibility that excess urinary urate concentration and urate crystal precipitation, and associated tubular injury may partly contribute to both nephrolithiasis and renal dysfunction in RHUC. Urinary b_2_-microglobulin was increased in the present case. Our previous study demonstrated that patients with renal hypouricemia had significantly higher urinary β_2_-microglobulin levels than patients with gout or normouricemia, and that urinary β_2_-microglobulin levels correlated with the fractional excretion of uric acid. These findings suggested that renal hypouricemia may be associated with proximal tubular dysfunction in addition to urolithiasis [26]. Consistent with these observations, the present patient showed markedly increased urinary urate excretion accompanied by elevated urinary β_2_-microglobulin. Furthermore, she had CKD G3b with a gradual decline in renal function. These findings raise the possibility that persistent excessive urinary urate excretion in RHUC1 may be associated with chronic tubular injury, although a causal relationship between RHUC1 and CKD cannot be established from a single case.

In conclusion, the present case suggests that the coexistence of rare, functionally impaired *URAT1* variants, A209V and F379L, can result in clinically overt RHUC with severe hypouricemia and excessive urinary uric acid excretion. Although allelic phasing was not performed, the clinical phenotype was consistent with presumed compound heterozygous URAT1 dysfunction. The coexistence of nephrolithiasis and CKD raises the possibility that excessive urinary urate excretion and stone formation may contribute to renal dysfunction. Furthermore, the clinical response to potassium citrate suggests that urinary alkalization may be useful for managing stone-related urinary abnormalities in patients with RHUC.
